# Treatment of diabetic wound based on hypoglycemic and antioxidant

**DOI:** 10.3389/fbioe.2025.1706136

**Published:** 2026-01-05

**Authors:** Xinyan Li, Yang Tan, Jie Li, Wei Sun, Yingshuai Wang, Yong Zhang

**Affiliations:** 1 School of Clinical Medicine, Shandong Second Medical University, Weifang, China; 2 Department of Internal Medicine, Affiliated Hospital of Shandong Second Medical University, Weifang, China; 3 Department of Neurology, The Third Affiliated Hospital of Southern Medical University, Guangzhou, Guangdong, China; 4 Department of Intensive Care Unit, Affiliated Hospital of Shandong Second Medical University, School of Clinical Medicine, Shandong Second Medical University, Weifang, China; 5 Department of Bioscience and Technology, Shandong Second Medical University, Weifang, China

**Keywords:** antioxidant, diabetic ulcers, drug delivery systems, hypoglycemic, nanomedicines

## Abstract

Diabetic ulcers are among the severe complications of diabetes. The accumulation of hyperglycemia and reactive oxygen species (ROS) in these ulcers significantly delays wound healing. Nanomedicine offers promising advantages for targeted drug delivery and enhanced therapeutic efficacy in treating diabetic ulcers associated with hyperglycemia and ROS. Effective treatment relies on the accessibility of suitable medications and wound dressings. This review summarizes current therapeutic strategies for diabetic skin injuries, focusing on two main categories: drugs (hypoglycemic agents and antioxidants) and drug delivery systems (hydrogels, nanofibers, and microneedle patches). By systematically analyzing these approaches, this review aims to inspire and guide the development of advanced nanomedicines for diabetic wound healing.

## 1 Introduction

Diabetic ulcers, one of the most severe complications of diabetes, have seen a significant rise in prevalence and incidence in recent years, becoming a major global health challenge (Li et al., 2020; Zhao et al., 2021). Elevated glucose levels and excessive reactive oxygen species (ROS) in the wound microenvironment are two key factors hindering effective healing.

Hyperglycemia markedly enhances the production of Advanced Glycation End-products (AGEs), which are stable compounds generated through non-enzymatic reactions between sugars and proteins. By binding to the Receptor for Advanced Glycation End-products (RAGE), AGEs trigger oxidative stress and stimulate the release of proinflammatory cytokines, directly contributing to the accumulation of high ROS levels and aggravating oxidative damage (Indyk et al., 2021). Moreover, high glucose levels impair tissue repair by disrupting the function of skin cells, including keratinocytes, fibroblasts, and vascular endothelial cells. This dysfunction leads to reduced cell proliferation, diminished migratory capacity, and decreased collagen synthesis (Li et al., 2017). Additionally, a high-sugar environment also promotes the growth of pathogenic bacteria, forming biofilms at the wound site; this delays wound healing and increases the risk of infection (Pitocco et al., 2019). Thus, proper glycemic control is essential for promoting the efficient healing of diabetic ulcers.

Another important reason for the difficulty in healing diabetic ulcers is hyperglycemia-induced oxidative stress. In physiological conditions, ROS function as signaling molecules to maintain redox homeostasis. A moderate increase can induce an adaptive immune response, promoting overall health (Sies and Jones, 2020; Sies et al., 2022). However, in diabetic wounds, the persistent hyperglycemic environment triggers mitochondrial dysfunction, leading to excessive ROS production. This excess ROS not only directly damages cell membranes, proteins, and DNA, but also activates pro-inflammatory signaling pathways (such as NF-κB), inhibits angiogenesis, and impedes the migration and proliferation of fibroblasts and keratinocytes. Consequently, this delays wound healing (Krzyszczyk et al., 2018; Gan et al., 2019). The accumulation of ROS in wounds triggers intense inflammatory responses (Zhao et al., 2020), inhibits the phagocytic function of macrophages, and impedes macrophage polarization from the pro-inflammatory M1 to the anti-inflammatory M2 phenotype. This leads to persistent inflammation, thus contributing to delayed wound healing. Therefore, regulating ROS levels and alleviating oxidative stress are important strategies for promoting the healing of diabetic ulcers.

The efficacy of current clinical treatments (such as debridement, wound dressings, growth factor application, skin flap transplantation, *etc.*) is limited, often resulting in suboptimal wound healing outcomes. There is an urgent need to develop novel therapeutic strategies to promote effective repair of diabetic ulcers.

In recent years, nanomedicine has demonstrated tremendous potential in treating diabetes due to its unique physicochemical properties and targeted delivery capabilities. Its capabilities for controlled release and targeted delivery ensure stable, long-term drug release during treatment, enhancing both therapeutic continuity and precision (Shi et al., 2017). Certain nanomaterials, such as metal-organic frameworks (MOFs) (Xiong et al., 2024) and carbon dots (Yan et al., 2025), possess glucose oxidase (GOx)-like and superoxide dismutase (SOD)-like activities. These properties enable them to enhance glucose uptake and consumption while simultaneously scavenging ROS and reducing oxidative stress. Drug delivery systems like hydrogels and nanofibers maintains wound moisture, protect against microbial infection, and absorb exudate, thereby promoting wound healing. Based on this, we summarize the research progress of nanodrugs and nanocarriers with hypoglycemic and antioxidant effects in treating diabetic ulcers. This review provides guidance for the future treatment of this disease with nanomedicine.

## 2 Pharmacological management strategies for diabetic ulcers

### 2.1 Nanoparticle-based targeted therapy for hyperglycemia

Currently, pharmacotherapy and insulin therapy are the primary clinical approaches for regulating blood glucose levels. Insulin therapy, which requires frequent subcutaneous injections, carries a risk of hypoglycemia. Oral medications, while convenient, often have a short duration of action. Encapsulating insulin in nanoparticles for oral delivery not only maintains its stability but also enhances intestinal absorption and bioavailability (Song et al., 2021), leading to a significant reduction in blood glucose levels. In addition, insulin regulates protein synthesis and promotes tissue repair (Regina and Tong, 2025). Similarly, combining metformin (MTF) with nanoparticles addresses the drug’s low permeability. The preparation of Metformin Nanostructured Lipid Carrier (MTF-NLC) improves drug permeability and enhances anti-inflammatory properties, thereby protecting target organs (Qushawy et al., 2025). Likewise, formulating systemic hypoglycemic drugs with nanomaterials improves drug utilization, enables sustained blood glucose control, and mitigates target organ damage.

**FIGURE 1:**
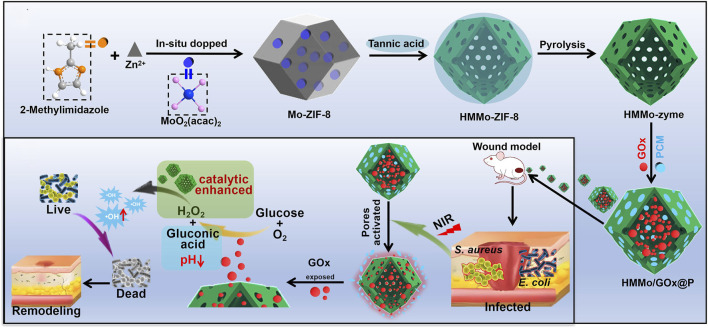
Synthetic schematic diagram of HMMo/GOx@P (Wang et al., 2025b).

In addition, nanomedicine-based local hypoglycemic strategies provide a new approach for the efficient repair of diabetic skin ulcers. In recent years, researchers have discovered that GOx and nanomaterials possessing redox enzyme-like properties catalyzes the conversion of glucose into gluconic acid and hydrogen peroxide (H_2_O_2_). By reducing the glucose concentration at the ulcer site, this process can promote wound healing (Zhang et al., 2018; Chen S. et al., 2025). Research has shown that hollow mesoporous molybdenum single-atom nanozymes (HMMo-zyme) can be utilized to encapsulate GOx, forming the HMMo/GOx@P system (as shown in Figure 1) (Wang et al., 2025b). This system leverages near-infrared (NIR) irradiation to cleave GOx, enabling continuous conversion of glucose to gluconic acid. Furthermore, studies have demonstrated that iron sulfide nanoparticle (FeSNP)-loaded ultrasmall gold nanoclusters (AuNCs) can form a cascaded nanozyme system (FeS@Au) (Yin et al., 2025). In this system, AuNCs exhibit glucose oxidase-like activity, catalyzing the conversion of glucose into gluconic acid and hydrogen peroxide (H_2_O_2_).

For diabetic ulcers, the glucose-depleting nanoagent consumes glucose at the local wound site and exerts antibacterial effects by generating toxic hydroxyl radicals through a cascade reaction, offering a novel strategy for efficient management of diabetic ulcers.

### 2.2 Antioxidant therapeutic drugs

ROS play a crucial role in wound healing, with appropriate concentrations of ROS contributing to accelerated healing. Clinically, antioxidants are commonly used to eliminate excess ROS, thereby promoting wound recovery (Peng et al., 2021). In addition to traditional antioxidant agents such as resveratrol (Res), curcumin, quercetin, and vitamin C (Wu et al., 2023; Pan et al., 2025), various nanomaterials have been identified for their antioxidant properties. These nanomaterials can reduce oxidative stress (Wang et al., 2025a), enhance ROS scavenging efficiency, and significantly promote wound healing.

#### 2.2.1 Nanozymes

Nanozymes are a class of materials that combine the unique properties of nanomaterials with enzyme-like catalytic functions (Ding et al., 2020). Compared to their natural enzyme counterparts, nanozymes exhibit enhanced stability and have been extensively studied in recent years (Huang et al., 2019; Wang et al., 2021).

Studies have shown that amorphous Ru@CoSe nanosheets can efficiently scavenge ROS due to their antioxidant enzyme-like activity (Deng et al., 2023). The novel multifunctional PtCuTe nanosheets also exhibit excellent catalytic performance and high efficiency in elimination ROS, making them promising materials for the treatment of diabetic ulcers (Guo et al., 2024). Moreover, a new bioactive nanozyme, cobalt-doped nanoglass (CoNZ), has been reported to exert antioxidant effects, although its mechanism involving the release of hydrogen peroxide (H_2_O_2_) warrants further investigation (Mandakhbayar et al., 2024). Research has also revealed that high-density platinum nanoparticle assemblies (PNAs) possess catalase (CAT) and peroxidase (POD) mimicking antioxidant activity. Under ultrasound (US) stimulation, PNAs can additionally induce glutathione (GSH) production, thereby further enhancing ROS scavenging (Zhang F. et al., 2023). The development of these nanozymes opens up new material pathways for promoting the healing of diabetic infected wounds through antioxidant strategies. Recent research has demonstrated that the FC/ZAG multifunctional wound dressing exhibits glucose-lowering and antibacterial effects through the cascade catalytic effect of ZAG nanozymes, and demonstrates a high safety profile (Wang X. et al., 2025).

#### 2.2.2 Hydrogen-producing nanomaterials

Hydrogen possesses a unique ability to selectively reduce inflammation and oxidation. Unlike common antioxidants, molecular hydrogen specifically neutralizes highly toxic hydroxyl radicals and peroxynitrite (Chen H. et al., 2022). Additionally, compared to other anti-inflammatory gaseous molecules such as CO, NO, and H_2_S, hydrogen demonstrates a superior safety profile (Nakao et al., 2009). However, traditional administration methods face significant limitations in terms of delivery efficiency and sustained efficacy due to hydrogen’s high diffusivity and low solubility. Integrating gas therapy with nanomedicine holds great promise for enabling the long-term, controlled release of hydrogen at lesion sites. Nanoscale hydrogen-releasing agents not only facilitate the loading and delivery of hydrogen donors and carriers but also utilize passive targeting to enhance hydrogen accumulation at the lesion sites.

**FIGURE 2:**
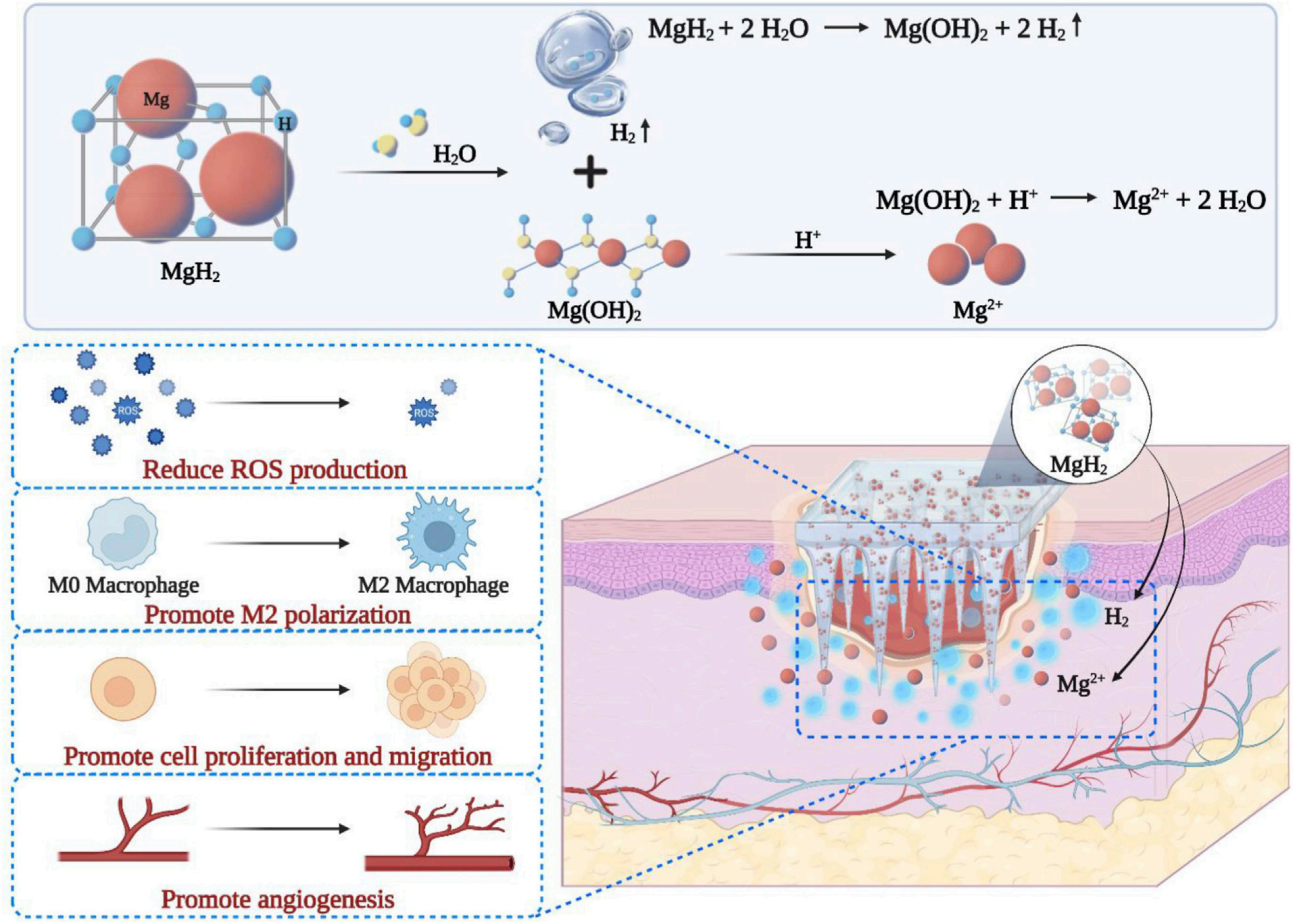
Schematic diagram of MgH_2_ microneedle patch and its functional mechanism (Wang et al., 2023).

Studies have shown that a microbial-hydrogel system can continuously produce hydrogen gas for up to 60 h (Chen H. et al., 2022). Building on this, a study developed microneedle patches (MN-MgH_2_) loaded with MgH_2_ (as shown in Figure 2) (Wang et al., 2023). After reacting with body fluids, these patches generate hydrogen gas (H_2_) and magnesium ions (Mg^2+^), with H_2_ reducing the production of ROS, thereby altering the pathological microenvironment of diabetes. Meanwhile, another study employed a photocatalytic system containing hydrogenated titanium oxide nanorods (HTON) to treat diabetic wounds (Chen S. et al., 2022). This system continuously generates hydrogen, effectively suppressing AGEs synthesis and receptor expression, thereby inhibiting skin cell apoptosis and promoting the proliferation and migration of skin cells.

#### 2.2.3 Nanoparticles

Nanoparticles (NPs) are synthesized from various organic and inorganic materials, such as nanoscale polysaccharides, proteins, lipids, DNA, carbon nanotubes, mesoporous materials, and more. They can deliver drugs to target sites through chemical conjugation or physical encapsulation (Debele and Park, 2022). Additionally, NPs have a small size, stable properties, and are easily absorbed by cells, making them highly effective for controlling drug delivery and release (Qin et al., 2022).

Recent studies have developed multiple nano-strategies for diabetic wound healing. Zirconium-based mesoporous MOFs with catalase-like activity were combined with SOD to form S@M@H NPs, which scavenge ROS via a cascade reaction, thereby inhibiting fibroblast senescence and ferroptosis to accelerate healing (Li et al., 2024). Separately, a rigid cerium oxide nanoparticle named CERATE eliminates and suppresses ROS production catalytically (Kim Y. G. et al., 2025). Meanwhile, another team created self-assembled CIZ nanoparticles and incorporated them into a hydrogel (CIZ@G), forming a system with potent antioxidant and anti-inflammatory activities (Wu et al., 2025).

Antioxidant nanomedicines are generally classified into three categories: First, nanozymes, which exhibit enzyme-like activity to exert antioxidant effects; second, hydrogen-producing nanomedicines, which scavenge local ROS by continuously producing hydrogen; and third, nanoparticles loaded with organic or inorganic materials, which can inhibit oxidative stress through multiple pathways. These nanodrugs demonstrate superior therapeutic effects compared to traditional antioxidant medications. However, challenges remain in ensuring the stability and catalytic activity of nanozymes, while maintaining the sustainability of hydrogen production is a critical issue. Additionally, due to the material diversity inherent in nanoparticles, both their preparation and testing processes are complex. Based on their distinct mechanisms of action, these drugs have been systematically classified (as shown in Table 1).

**TABLE 1:** The classification of hypoglycemic and antioxidant drugs.

	Classify	Type	Drugs	Size	Mouse model	ROS measurements	Cell viability assays	Photothermal effect	Antibacterial	Pro-angiogenic	Pathway	Efficiency	Cite
Antioxidant	Traditional medicines		Res	/	STZ induction	DCFH-DA	CCK-8	/	/	✓	PI3K-AKT-Nrf2	Enhancements	Pan et al. (2025)
	Nanomedicine	Nanozymes	PtCuTe	/	/	CellROX	/	/	✓	✓	/	8 days heals 91%	Guo et al. (2024a)
			CoNZ	54.8(±5) nm	STZ induction	10mMc-H_2_ DCFH-DA	CCK-8	/	/	✓	/	14 days heals 94.5%	Mandakhbayar et al. (2024)
			PNAs	221 nm	/	/	/	/	/	✓	/	9 days heals 95%	Zhang et al. (2023a)
		Hydrogen-Producing Nanomaterials	MN-MgH_2_	8.1 μm	db/db	DCFH-DA	CCK-8	/	/	✓	/	10 days to complete	Wang (2023)
			HTON	Diameter 20 nm Length 100- 400 nm	STZ induction	/	CCK/8	✓	/	✓	AGE-RAGE	14 days to complete	Chen et al. (2022a)
		Nanoparticles	S@M@H NP	205 nm	STZ induction	DCFH-DA	CCK-8	/	/	✓	cGAS-STING	12 days heals 99.2%	Li et al. (2024)
			CIZ	210.4 ± 30.3 nm	STZ induction	DCFH-DA DPPH	/	✓	✓	✓	/	14 days heals 99.1%∼99.7%	Wu et al. (2025)
Hypoglycemic	Overall Hypoglycemic		EUAC-Ins	210 ± 35.5 nm	STZ induction	/	/	/	/	/	/	/	Song et al. (2021)
			MTF-NLC	247.72 ± 5.74-503.23 ± 7.26 nm	STZ induction	/	/	/	/	/	/	/	Qushawy et al. (2025)
	Local wound hypoglycemia		HMMo/GOx@P	/	STZ induction	/	CCK-8	✓	✓	/	/	7 days heals 79%	Wang et al. (2025b)
			FeS@Au	75 nm	STZ induction	DCFH-DA	CCK-8	/	✓	✓	HIF-1 signaling	10 days to complete	Yin et al. (2025)
Hypoglycemic and Antioxidant			Fe_3_O_4_-GOx	12.7 ± 2.5 nm	db/db	/	/	/	√	/	/	15 days to complete	Du et al. (2022)
			Zn-DHM NPs	5 nm	STZ induction	ABTS⋅+, DPPH⋅, PTIO⋅,TMB	CCK-8	/	/	✓	HIF-1 signaling, AGE-RAGE signaling, IL-17 signaling	14 days heals 99.70%	Zhang et al. (2025b)

### 2.3 Drugs with synergistic effects of hypoglycemic and antioxidant actions

**FIGURE 3:**
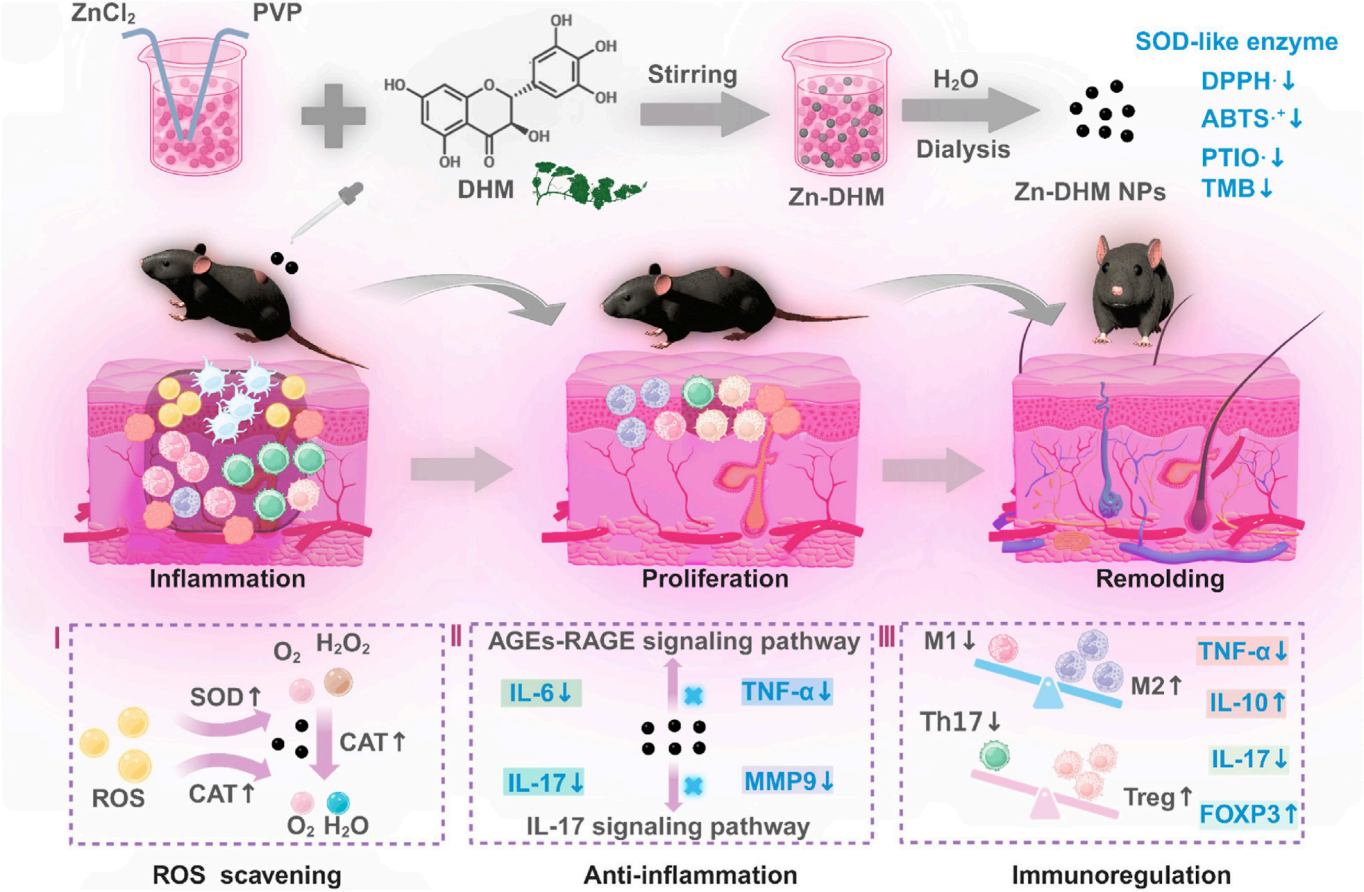
The schematic diagram illustrating the mechanism of Zn-DHM NPs in promoting diabetic wound healing (Zhang S. et al., 2025).

Studies have shown that nanonzymes composed of iron oxide nanoparticles (Fe_3_O_4_) encapsulating GOx exhibit the activities of GOx, catalase (CAT), and peroxidase (POD). These nanonzymes catalyze GOx/POD and GOx/CAT cascade reactions triggered by glucose and effectively scavenge excess reactive oxygen species (ROS) (Du et al., 2022). Another study developed cerium oxide (CeO_2_) nanoparticles encapsulated in zeolitic imidazolate framework-8 (ZIF-8) with absorbed glucose oxidase (GOx), forming a composite nanonzyme designated as [(ZIF-8@CeO_2_)@GOx, zcg] (Zhang X. et al., 2025). In this system, GOx catalyzes glucose oxidation, thereby triggering a cascade reaction for antioxidation and anti-glycation. A recent study discovered a novel metal-polyphenol nanonzyme, Zn-DHM NPs (Zhang S. et al., 2025), which enhances ROS scavenging by upregulating intracellular levels of superoxide dismutase (SOD) and catalase (CAT) (as shown in Figure 3).

Nanodrugs with hypoglycemic and antioxidant effects primarily function by mimicking enzymatic activity to catalyze key reactions. Additionally, they reduce the production of inflammatory mediators and promote angiogenesis, significantly enhancing wound healing in diabetic wounds.

## 3 Drug delivery systems

### 3.1 Hydrogels

Hydrogels exhibit properties including hemostasis, anti-inflammation, antibacterial activity, and the promotion of cell proliferation, while also facilitating cellular signal transduction within tissues (Lei and Fan, 2022). Their characteristic porous structure promotes blood cell adhesion, thereby enhancing coagulation (Liu et al., 2022). Moreover, their swelling capacity allows for effective absorption of wound exudate, maintenance of a moist wound environment, and controlled drug release—collectively contributing to accelerated wound healing (Yang et al., 2024). Currently, hydrogels offer distinct advantages in wound management applications.

**FIGURE 4:**
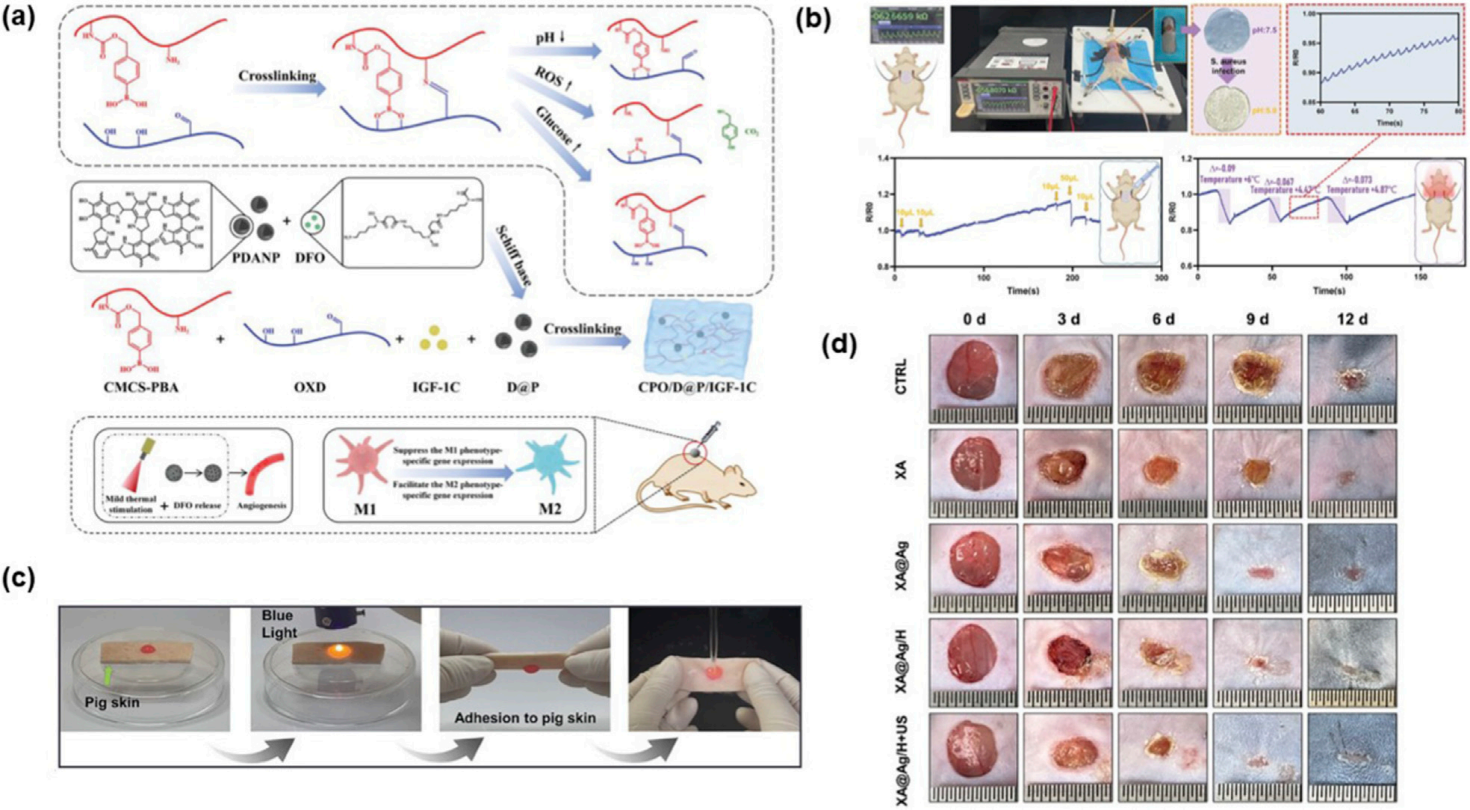
**(a)** Synthesis diagram of CPO/D@P/IGF-1C hydrogel and mechanism schematic diagram for promoting diabetic wound healing by hydrogel combined with mild thermal stimulation (Dai et al., 2024). **(b)** N-P/B/SH/[TA (siRNA)@BSA] Hydrogel Monitors Heartbeat, Wound Exudate, and Temperature Changes (Zong et al., 2024). **(c)** The formation process and adhesion of N-P/B/SH/[TA (siRNA)@BSA] hydrogel (Zong et al., 2024). **(d)** Typical photographs showing the wound healing progression trajectory in diabetic mouse models treated with XA@Ag/H + US hydrogel (Bei et al., 2024).

To promote diabetic wound healing, an injectable polymeric hydrogel has been synthesized, enabling sustained release of Total Glucosides of Peony (TGP) (Zhang Y. et al., 2023). The CPO/D@P/IGF-1C hydrogel (as shown in Figure 4a) demonstrates sustained drug release, excellent photothermal effects, and outstanding antioxidant, antibacterial, and anti-inflammatory activities (Dai et al., 2024). Another study developed a nanocomposite N-P/B/SH/[TA (siRNA)@BSA] hydrogel (Lei et al., 2024), which exhibits high adhesiveness and shape adaptability while also monitoring changes in pH, temperature, and wound exudate during the healing process (as shown in Figures 4b,c). A separate study fabricated an ultrasound-responsive hydrogel (XA@Ag/H) (Zong et al., 2024), that enables deep penetration into wounds (exceeding 400 μm) and facilitates wound healing (as shown in Figure 4d). Additionally, the multifunctional hydrogel PAN/Ag-PLG has been reported to combine strong tissue adhesive with easy detachment properties (Bei et al., 2024). More recently, a composite hydrogel (O-GG/HA@EM) has been shown to effectively alleviate oxidative stress and hypoxic microenvironments at the wound site while inhibiting bacterial infection (Luo et al., 2025). Latest research has developed a glucose-responsive smart hydrogel, loaded with honokiol and metformin, which integrates antibacterial and antioxidant properties with its defining glucose-triggered drug release capability. (Zhai et al., 2025).

Hydrogels are known for their excellent mechanical properties, good biocompatibility, and antibacterial effects. They can also be used to deliver drugs in a sustained-release manner. Some hydrogels even offer monitoring capabilities. However, hydrogels with such functionality often exhibit poor mechanical strength. Moreover, their strong adhesiveness can make removal difficult, and since most hydrogels are not fully degradable, they may leave residue at the wound site.

### 3.2 Nanofibers

Nanofibers, typically fabricated via electrospinning, offer enhanced moisture transport, improved cell adhesion, and an anisotropic structure. When applied to diabetic wounds, nanofibers can optimize cellular sensitivity to structural signals, enhance cell migration, and promote efficient wound healing (Kim K. et al., 2025).

Studies have shown that near-infrared (NIR)-responsive MoS_2_@Pd nanonzymes were further synthesized PLGA/MoS_2_@Pd nanofiber membranes (Chen Z. et al., 2025). These membranes effectively scavenge reactive oxygen species (ROS) and reduce oxidative stress damage in fibroblasts. Another study developed poly (L-lactic acid) (PLLA) nanofiber sutures loaded with a Salvia miltiorrhiza-Pueraria lobata (SRHC) herbal complex (Li et al., 2025). The addition of SRHC significantly enhanced the tensile and knot strength of the sutures, while also improving its antioxidant and anti-inflammatory properties. Furthermore, a novel nanofiber pad (S3) not only exhibited antibacterial effects (Ghosh et al., 2025) but also promoted wound contraction by reducing blood glucose levels, alleviating hepatotoxicity, and inhibits oxidative stress biomarkers. Another study found that combining chitosan (CS) with polycaprolactone/gelatin (PG) nanofibers resulted in the creation of a P/G-CS-OI membrane (He et al., 2024). This membrane promotes macrophage polarization towards the repair phenotype and mitigates inflammatory responses.

Both nanofibers and hydrogels share characteristics such as maintaining tissue moisture, enabling targeted drug delivery, and exhibiting good mechanical properties. However, their adhesion to tissues is generally modest.

### 3.3 Microneedle patches

Microneedles were first introduced in 1976 to facilitate drug delivery. They range in length from 25 μm to 2,000 μm and are much sharper than the tips of hypodermic needles. As a result, microneedles penetrate the stratum corneum, creating micro-channels for drug delivery, which significantly enhances delivery efficiency (Ma and Wu, 2017).

Studies have shown that microneedles (MNs) with dual fast and slow degradation characteristics can accelerate angiogenesis and tissue regeneration, and enable sustained release of desferrioxamine (DFO) and dopamine (Ran et al., 2024). Another study encapsulated Au-CMS nanoparticles (NPs) into microneedle patches (MNs) (Shan et al., 2022), utilizing near-infrared-II photothermal effects to achieve *in situ* glucose consumption and bacterial killing. Furthermore, research has proposed loading core-shell Ag@MSN@CeO_2_ nanoparticles (NPs) into soluble microneedle patches (MNs) to achieve deep tissue penetration for efficient drug delivery (Yu et al., 2024).

Microneedle patches differ most significantly from the other two systems in that their sharp tips allow them to deliver drugs deep into wounds, offering excellent tissue penetration and enabling drug action at greater depths. However, their inability to stretch and adapt to the wound makes it difficult for them to conform precisely to irregular wound shapes, which remains a current challenge. The diversity of drug delivery systems is summarized in Tables 2, 3 presents an integration of the different medication forms.

**TABLE 2:** Classification of drug delivery systems.

	Classify	Drugs	Cross-linking keys	Adhesion to tissues	Function	Characteristic	Efficiency	Cite
Hydrogel	Natural hydrogel	CPO/D@P/IGF-1	covalent bond	covalent bond	Anti-inflammatory, Antibacterial	Injectability, Degradability, Photothermal stability, Sustained-release drug performance, Flexibility	13 days to complete	Dai et al. (2024)
		XA@Ag/H	covalent bond, hydrogen bond	/	Hemostatic, Antibacterial, Biofilm elimination	Injectable, Ultrasound Responsive, High permeability	12 days to complete	Zong et al. (2024)
		O-GG/HA@EM	covalent bond	covalent bond, hydrogen bond	Antibacterial, Hemostatic	Injectability, Dynamic response characteristics, Sustained drug release performance, Flexibility	14 days heals 92.4%	Luo et al. (2025)
	Synthetic hydrogels	N-P/B/SH/[TA(siRNA)@BSA]	covalent bond, hydrogen bond	hydrogen bond/disulfide bonds	Anti-inflammatory and Immunomodulatory, Antibacterial, Hemostasis	Monitorability, Degradability, Good conductivity	10 days to complete	Lei et al. (2024)
		PAN/Ag-PLG multifunctional	Non-covalent bonds	covalent bond, hydrogen bond	Antibacterial, Hemostatic, Anti-inflammatory	Adhesion, Easy peelability, Flexibility	21 days to complete	Bei et al. (2024)
Nanofibers	Electrospinning	PLGA/MoS_2_@Pd nanofiber membranes	/	/	Antibacterial, Anti-inflammatory, Hemostasis	Photothermal properties	14 days heals 98.563%	Chen et al. (2025b)
		S3	covalent bond, hydrogen bond	/	Antibacterial, Prevent microbial penetration and biofilm formation	Hydrophilic, Antimicrobial film efficacy	Enhancements	Ghosh et al. (2025)
	Electrospinning and hot drawing processes	PLLA nanosutures for SRHC	/	/	Anti-inflammatory	Flexibility	/	Li et al. (2025)
Microneedle patch		Dual-module MNs	Physical cross-linking, covalent bond	/	Anti-inflammatory, Hemostasis	Degradability, Enhancing drug delivery, Low invasiveness	14 days to complete	Ran et al. (2024)
		Au-CMS@ MNs	/	/	Antibacterial	Photothermal effect, Enzyme-like activity	9 days heals 90%	Shan et al. (2022)

**TABLE 3:** Dosage form comparison.

Ingredients	Dosage form	Drugs	Mechanism	Efficacy	Cite
Cu	hydrogel	Cu-MOF/GOX	cascade reaction	11 days heals 92.02%	Chen et al. (2025a)
	PVA	PtCuTe	upregulating angiogenic factors	8 days heals 91%	Guo et al. (2024)
DFO, DA	hydrogel	CPO/D@P/IGF-1C	up-regulating the expression of anti-inflammatory factors and down-regulating the expression of pro-inflammatory factors	13 days to complete	Dai et al. (2024)
	microneedles	The dual-modular MNs	the transfection of miRNA, inhibits the nuclear factor (NF)-𝜅B signaling pathway	14 days to complete	Ran et al. (2024)
	cryogels	GA/HD/MDP	/	21 days to complete	Wang et al. (2025a)
CeO_2_	hydrogel	CERATE	upregulated EGF and PDGF-BB	/	Kim et al. (2025b)
	microneedles	MN@Ag@MSN@CeO2	upregulated HIF-1α, VEGF, and PDGF	12 days to complete	Yu et al. (2024)
MOF	nanoparticles	S@M@H NPs	cGAS-STING	12 days heals 99.2%	Li et al. (2024)
	hydrogel	Cu-MOF/GOX	cascade reaction	11 days heals 92.02%	Chen et al. (2025a)
Au	microneedles	Au-CMS NSs	enzyme-like activity	9 days heals 90.0%	Shan et al. (2022)
	nanozymes	FeS@Au	cascade reaction, HIF-1 signaling, VEGF signaling	/	Yin et al. (2025)
Zn	hydrogel	CIZ@G	Il-10, CD206, CD31, α-SMA and VEGF were increased, TNF-α and CD86 were inhibited,	14 days heals 99.1%-99.7%	Wu et al. (2025)
	PVP	ZN-DHM NPs	cascade reaction, HIF-1 signaling, AGE-RAGE signaling, IL-17 signaling	14 days heals 99.7%	Zhang et al. (2025a)

## 4 Conclusion

The rising prevalence of diabetic ulcers necessitates multidisciplinary strategies, as monotherapeutic nanomedicines often fail to modulate the complex wound microenvironment. A promising solution involves engineering multifunctional nanomedicines through the integration of nanomaterials with catalytic enzymes, hydrogen-releasing agents, and inorganic ions. This approach enables precise regulation of the wound microenvironment, thereby enhancing therapeutic efficacy and safety. Furthermore, embedding these nanodrugs into platforms such as antibacterial hydrogels or permeable microneedles can provide pro-angiogenic, antioxidant, and anti-inflammatory functions, significantly improving healing outcomes.

Despite its promise for diabetic ulcer treatment, nanomedicine faces challenges in preparation and safety. Future work will therefore focus on creating more stable, safe, and specific nanomedicines through material integration. This advancement is crucial for overcoming the technical and biological barriers to clinical translation.
